# Organizational models for acute gastrointestinal bleeding: a systematic review of hospital networks, multidisciplinary care and bloodless programs (2015–2025)

**DOI:** 10.3389/fmed.2026.1881585

**Published:** 2026-07-02

**Authors:** Sungat Otegen, Lyudmila S. Yermukhanova, Abylai Baimakhanov, Negar Ashuri, Aruzhan Otegenova Muratkyzy, Aidyn Armanuly Zhylgeldy, Murat Jakanov, Gulbanu Arynova, Alireza Afshar

**Affiliations:** 1 Department of Public Health and Public Health Care, West-Kazakhstan Marat Ospanov Medical University, Aktobe, Kazakhstan; 2 Faculty of Postgraduate Education, Asfendiyarov Kazakh National Medical University, Almaty, Kazakhstan; 3 Student Research Committee, Bushehr University of Medical Sciences, Bushehr, Iran; 4 Department of General Medicine, Asfendiyarov Kazakh National Medical University, Almaty, Kazakhstan; 5 Department of the Neurosurgery, West-Kazakhstan Marat Ospanov Medical University, Aktobe, Kazakhstan; 6 Department of General Surgery, West Kazakhstan Medical University named after Marat Ospanov, Aktobe, Kazakhstan; 7 Department of Epidemiology, Biostatistics and Evidence-Based Medicine, Al-Farabi Kazakh National University, Almaty, Kazakhstan

**Keywords:** blood transfusion, clinical pathways, delivery of health care, gastrointestinal hemorrhage, patient care team

## Abstract

**Background:**

Acute gastrointestinal bleeding is common, yet evidence on how best to organize hospital services for these emergencies is limited. To synthesize evidence on organizational models for delivery of care to adults with acute gastrointestinal bleeding.

**Methods:**

We systematically searched four databases (2015–2025) for studies evaluating organizational interventions in hospitalized adults with upper or lower gastrointestinal bleeding. Eligible models included hub and spoke networks, multidisciplinary team (MDT) programs, structured care bundles and transfusion free pathways. Two reviewers screened records, extracted data and assessed risk of bias using ROBINS-I. Owing to heterogeneity of interventions and outcomes; we undertook narrative synthesis. Primary outcomes were mortality, rebleeding, readmission, length of stay and process measures.

**Results:**

Of 978 records identified, three studies met the inclusion criteria. One large cohort compared hub and spoke network hospitals with non network hospitals and found no difference in risk adjusted mortality, although gastroenterology wards and academic centers were associated with better survival. A single center MDT programmed for cirrhotic variceal bleeding reported lower mortality, reduced rebleeding, fewer readmissions and shorter stay. A bloodless medicine cohort showed that transfusion free management achieved low mortality despite very low nadir hemoglobin. Reporting of quality of life and other patient reported outcomes was very rare. All studies were at serious risk of bias, with limited generalizability and no economic evaluation.

**Conclusion:**

Evidence on organizational models for acute gastrointestinal bleeding is sparse but suggests potential benefits of multidisciplinary and bloodless care programs, while network affiliation alone may be insufficient to improve outcomes.

## 1 Introduction

Acute gastrointestinal bleeding (GIB) remains one of the most frequent reasons for hospital admission and continues to impose a considerable clinical and economic burden (1). Population-based estimates suggest that upper GIB (UGIB) affects 60–160 people per 100,000 population each year, while lower GIB (LGIB) occurs in 20–87 per 100,000 (2, 3). In the United States, UGIB alone results in more than 300,000 hospitalizations annually and accounts for nearly 40% of admissions for digestive diseases (1, 4). The direct health-care costs of GIB exceed 2 billion USD per year (1), and one large administrative analysis estimated that the total cost of GIB-related hospitalizations approaches 5 billion USD when accounting for coding inaccuracies (5). Despite improvements in endoscopic and pharmacologic therapies, mortality rates for acute GIB remain concerning: case-fatality rates range from 3.5 to 14% (6), with population-based mortality between 0.9 and 9.8 deaths per 100,000 (2).

The burden of GIB is particularly pronounced in older adults and men. A nationwide Finnish cohort reported that crude incidence rates were 1.7 cases per 1,000 person-years for GIB, with age standardized rates higher in men than women and LGIB more common than UGIB (7). In the UK, the British Society of Gastroenterology audit found that UGIB has an incidence of approximately 134 per 100,000 population equivalent to one presentation every 6 min and a mortality rate approaching 10% (8). In addition to this high fatality, quality of care audits have repeatedly revealed deficiencies in risk assessment, resuscitation, and timely endoscopy (8). International guidelines therefore emphasize early haemodynamic stabilization, risk stratification using scores such as the Glasgow-Blatchford Score, restrictive transfusion strategies, proton-pump inhibitor infusion and endoscopy within 24 h (8, 9). For example, the American Family Physician review recommends upper endoscopy within 1 day of presentation, noting that non-variceal UGIB still carries an in-hospital mortality around 13%. However, there is debate about the benefits of urgent versus early endoscopy; a recent randomized trial and several observational studies found no mortality difference between endoscopy performed within 6 h versus within 24 h (10), highlighting the importance of coordinated care rather than timing alone.

Acute GIB management is inherently multidisciplinary. Patients often require simultaneous resuscitation, transfusion management, pharmacologic therapy, endoscopic intervention, and in selected cases interventional radiology or surgery (11). Efficient communication between emergency, gastroenterology, surgery and anesthesia teams can reduce delays and adverse events (3). Unfortunately, variations in hospital resources, specialty availability and organizational structure contribute to wide disparities in outcomes. A study of U. S. hospital performance metrics found that only 8.3% of institutions improved mortality, length of stay and complication rates simultaneously between 2016 and 2018, and the correlation between mortality and length of stay was weak (*r* = 0.22) (12). Such heterogeneity suggests that process of care factors and organizational models may substantially influence patient outcomes.

In response, several innovative delivery models have been proposed. Hub-and-spoke networks aim to centralize specialized endoscopy and offer structured referral pathways; they connect smaller “spoke” hospitals to a “hub” center with 24 h endoscopy and interventional radiology. Multidisciplinary team (MDT) care integrates gastroenterologists, hepatologists, anesthetists, nurses, dietitians and social workers to provide comprehensive management, particularly for complex cases such as variceal hemorrhage in cirrhotic patients. Transfusion-free protocols involve dedicated bloodless medicine units that accept patients who refuse blood products; they emphasize optimization of hemostasis and restrictive transfusion, often guided by iron supplementation, erythropoietin and point-of-care coagulation testing. Additionally, care bundles and communication protocols have been introduced to standardize early management; for instance, a multidisciplinary communication protocol implemented in a trauma center led to earlier endoscopy, reduced transfusion requirements, shorter length of stay and fewer readmissions (3, 13). Despite these innovations, the effects of organizational models on outcomes remain unclear, and previous reviews have focused predominantly on pharmacologic or endoscopic therapies rather than the structure of care delivery. Bloodless medicine programs were included because they constitute hospital-wide organizational interventions. Such programs require dedicated units with specialized staff, policies and protocols for haemostatic optimization, iron supplementation, erythropoiesis and point-of-care coagulation testing, and they are managed by multidisciplinary teams (14). These programs exemplify the patient blood management paradigm, which emphasizes restrictive transfusion and conservation of the patient’s own blood and is endorsed as a standard of care. Consequently, we considered the transfusion-free cohort to represent more than a disease-specific pathway and therefore relevant to our review (15). These models can be situated within a shared framework of organizational interventions that restructure the delivery of care. Hub-and-spoke networks operate at the regional level, concentrating expertise through centralization; MDT programs function at the hospital level by integrating gastroenterologists, hepatologists, anesthetists, nurses and other specialists to coordinate complex care; and transfusion-free programs represent protocol-driven, hospital-wide initiatives focused on patient blood management. Despite their differences, all three interventions aim to improve outcomes by addressing systemic barriers such as delayed access to specialists, fragmented communication and inconsistent transfusion practices (15).

Transfusion-free programs are not merely individual-level therapeutic strategies; they function as hospital-wide organizational models. Comprehensive patient-blood-management programs require institutions to implement pre-operative, intra-operative and post-operative safety measures and patient-tracking systems to avoid inadvertent transfusion (16). They emphasize evidence-based, multidisciplinary care that begins with initial patient evaluation and continues through the entire clinical pathway (17, 18). Effective implementation requires coordinated input from hematology, anesthesiology, surgery and gastroenterology services, highlighting the program’s organizational scope (15). In our conceptual framework, organizational interventions are defined as models that restructure how care is delivered across institutions; transfusion-free programs meet this definition because they depend on dedicated teams, hospital-level policies and system-wide protocols rather than a single therapeutic maneuver.

Given the substantial burden of GIB and the potential for organizational factors to improve outcomes, there is a need to synthesize evidence on care-delivery models. To our knowledge, no comprehensive systematic review has evaluated hub-and-spoke networks, multidisciplinary team programs or transfusion-free pathways for acute GIB. This study therefore aimed to assess the impact of organizational and care-delivery interventions on mortality, rebleeding, length of stay and resource use among adults with acute gastrointestinal bleeding. By appraising the available evidence on innovative service models, we sought to identify strategies that could be scaled to improve outcomes and inform guidelines. Although the literature on organizational models for GIB is sparse, our goal was to evaluate the impact of defined interventions on patient outcomes rather than to map the breadth of available evidence. Scoping reviews are appropriate when the aim is to explore the extent and nature of research activity (19), whereas systematic reviews are designed to synthesize and critically appraise evidence to guide practice. We therefore undertook a systematic review to apply rigorous inclusion criteria, assess methodological quality, and derive provisional conclusions about the effect of hub–and–spoke networks, MDT programs, and transfusion-free pathways.

## 2 Methods

### 2.1 Study design and registration

This systematic review was conducted according to the Preferred Reporting Items for Systematic reviews and Meta-Analyses (PRISMA) 2020 guidelines (20). The protocol was developed *a priori* and specified the research question, eligibility criteria, search strategy, data extraction and synthesis methods. The protocol was developed a priori but was not prospectively registered in a public registry such as PROSPERO due to time and resource constraints. We acknowledge this absence of registration as a limitation that may increase the risk of unreported protocol deviations.

### 2.2 Eligibility criteria

Studies were eligible if they met the following criteria: (1) included adults (≥18 years) with acute upper or lower gastrointestinal hemorrhage; (2) were conducted in hospital-based acute care settings such as emergency departments, inpatient wards, intensive care units or endoscopy units; (3) evaluated an organizational or care-delivery model (e.g., clinical pathways, protocols, multidisciplinary teams, hub-and-spoke networks, specialist units or transfusion-free programs); (4) reported patient-centered outcomes such as mortality, rebleeding, complications, quality of life, length of stay, readmission, time to endoscopy or transfusion use; (5) used randomized or observational designs including cohort, case–control, before-after or interrupted time-series studies with quantitative outcomes; and (6) were published in English, peer-reviewed journals between 1 January 2015 and 31 December 2025 and provided sufficient detail to classify the organizational model. Moreover, we grouped hub-and-spoke networks, MDT programs and transfusion-free pathways under the broad category of organizational models because each entails systematic changes to the structure or process of care delivery.

Exclusion criteria were: studies involving children or non-gastrointestinal bleeding; pharmacologic, device or procedural interventions without organizational changes; reports focusing only on surrogate or technical outcomes; outpatient-only, simulation-based or modeling studies without real patients; narrative or systematic reviews, guidelines, protocols, abstracts, theses or editorials without primary data; case reports or very small case series lacking structured pathways or comparator groups; non-human studies; non-English publications; studies with insufficient description of the organizational model; and duplicate or overlapping reports.

### 2.3 Data sources and search strategy

Search strategies were developed for each database (PubMed, Scopus, Embase and Web of Science) in consultation with an information specialist. Controlled vocabulary and free-text terms for gastrointestinal bleeding and care-delivery models were combined with Boolean operators. For example, the PubMed strategy combined terms for “gastrointestinal hemorrhage” or “gastrointestinal bleeding” with terms for healthcare delivery (“delivery of health care,” “service delivery,” “organization of care”) and care pathways or multidisciplinary teams, and applied limits for adults, English language and publication years 2015–2025. Similar concepts were translated to Scopus, Web of Science and Embase using database-specific syntax (Supplementary Tables S1–S4). Search filters were applied to retrieve studies published in the last 10 years, involving human adults, in English and, where available, open-access or free full-text articles. Reference lists of included studies and relevant reviews were screened to identify additional articles.

### 2.4 Study selection

**Figure 1:**
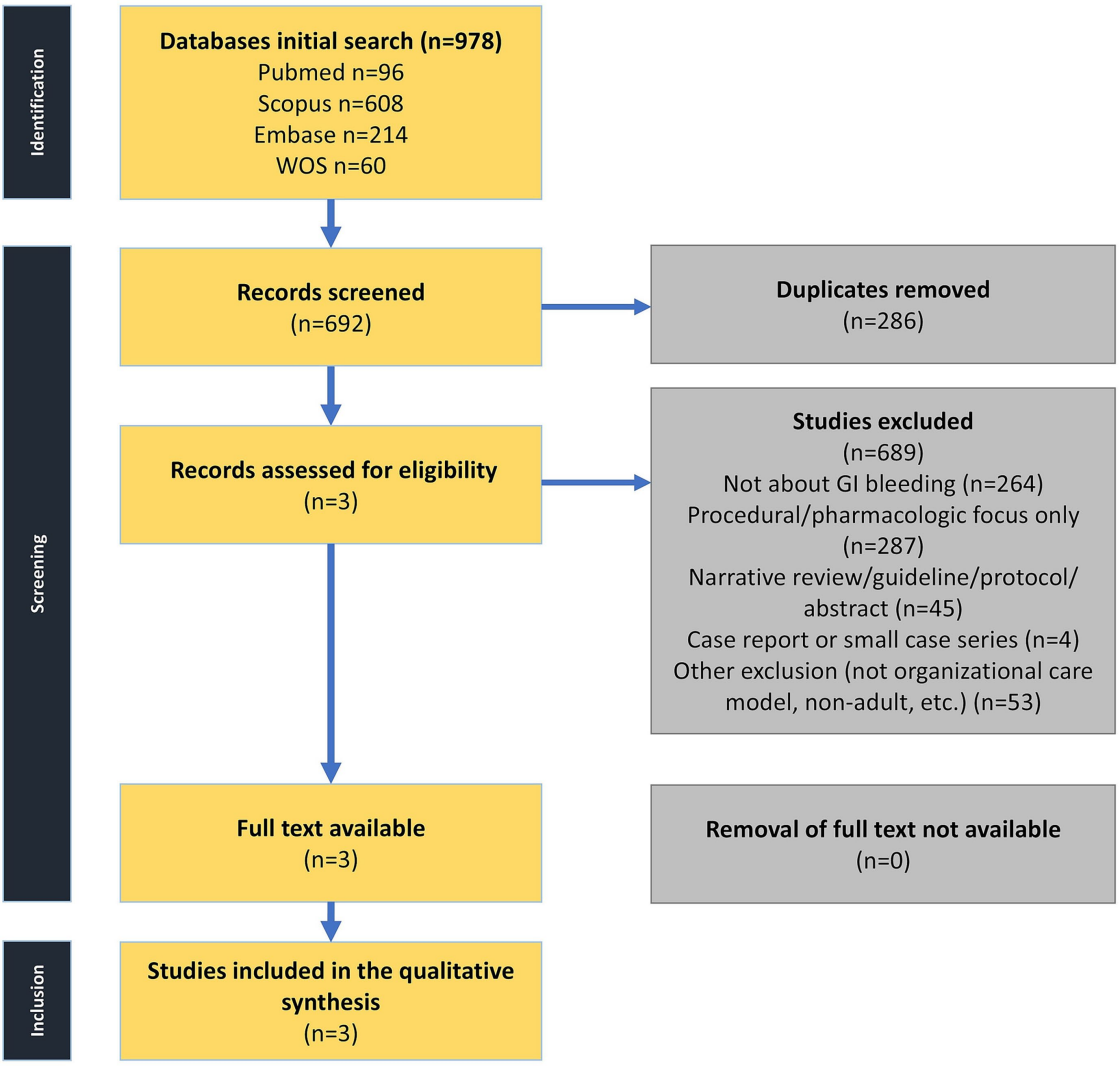
PRISMA flow diagram of study identification and selection for organizational models in acute gastrointestinal bleeding (2-15-2025).

All retrieved records were exported to reference management software, and duplicates were removed. Two reviewers independently screened titles and abstracts for relevance. Full texts were obtained for potentially eligible studies and assessed against the inclusion and exclusion criteria. Discrepancies were resolved by discussion and, where necessary, by consultation with a third reviewer. Reasons for exclusion were documented at each stage. The screening process is summarized in the PRISMA flow diagram (Figure 1).

### 2.5 Data extraction and quality assessment

A standardized data-extraction form was developed to collect information on study design, country and setting, sample size, patient characteristics, details of the organizational intervention, comparator, outcomes and key results. Two reviewers independently extracted data and cross-checked entries for accuracy. Study authors were contacted for clarification when necessary.

Risk of bias for non-randomized studies was assessed across the domains recommended by the Risk Of Bias In Non-randomized Studies-of Interventions (ROBINS-I) tool. The tool evaluates bias arising from participant selection, confounding, classification of interventions, deviations from intended interventions, missing data, measurement of outcomes and selection of reported results (21). For each domain, studies were judged as low, moderate, serious or critical risk of bias; an overall judgment reflected the highest risk level in any domain (Table 1). Although ROBINS-I version 2 with algorithmic judgments was introduced in 2025, we followed guidance available during the study period (21).

**Table 1:** Domain-specific ROBINS-I risk-of-bias assessment of included studies evaluating organizational care models for acute gastrointestinal bleeding.

Study	Selection bias	Confounding & control	Classification of intervention	Deviations from intended intervention	Outcome measurement	Missing data & follow up	Selection of reported results	Overall risk and justification
Marmo et al., 2021 (24)	Moderate: Prospective multicenter cohort enrolled all consecutive patients with UGIB admitted to 50 Italian hospitals over 2 years. However, hospitals self-selected into the network, so differences between in-network and out-of-network hospitals may persist.	Moderate: Multivariate logistic regression adjusted for clinical and organizational factors to control confounding. Still, unmeasured differences between network and non-network hospitals (e.g., staffing, resources) may remain.	Low: Exposure (network vs. non-network hospital) was determined at hospital level and clearly defined.	Low: Allocation to network or non-network hospitals was based on hospital membership and not under the control of participants or study investigators; patients were managed according to usual care at each hospital, so deviations from the organizational model were unlikely.	Low: Outcomes (mortality, rebleeding, length of stay [LOS], surgery) were prospectively collected and defined *a priori*.	Moderate: Data were collected via electronic CRFs with alerts for missing data, but analyses excluded records with missing values; the proportion of missing data is not reported.	Moderate: Major outcomes (mortality, rebleeding, LOS) were reported. However, not all process measures were provided and missing data were not fully characterized, posing a moderate risk of selective reporting.	Moderate: The study was large and prospectively collected, with clear outcome definitions and adjustment for confounders. Selection of hospitals and incomplete reporting of missing data temper confidence in causal inference.
Wang et al., 2021 (25)	Moderate: Single-center retrospective cohort included cirrhotic patients admitted between July 2015 and December 2019, but 56/321 were excluded and follow-up losses differed between groups (43 vs. 16).	Moderate: Allocation to Multidisciplinary team care (MDTC) vs. traditional care was based on admission date (before vs. after August 2017). Multivariable regression adjusted for the Model for End-Stage Liver Disease (MELD), Child-Pugh score and transfusion requirements, yet temporal improvements and other confounders may not be fully controlled.	Low: Intervention (MDTC vs. traditional care) was clearly defined and consistently applied over specific time periods.	Moderate: The report did not specify what proportion of patients received all MDT components or how adherence was monitored; deviations from the intended intervention may have occurred.	Moderate: Mortality and rebleeding were primary outcomes; quality of life measured with EQ-5D-5L. Outcome measurement was standard, but data collection was retrospective and depended on medical records and telephone follow-up.	Serious: Differential loss to follow-up (43 vs. 16 patients) could bias results. Exclusion of patients with incomplete records and those who died before treatment also risks selection bias.	Serious: The study reported mortality, rebleeding and quality of life but did not report all possible process outcomes (e.g., time to endoscopy). Exclusion of patients and incomplete reporting contribute to a serious risk of selective outcome reporting.	Serious: The before-after design is vulnerable to temporal confounding and attrition bias. Although adjustments were made, the non-random allocation and differential follow-up loss warrant caution.
Sharma et al., 2015 (14)	Moderate: Retrospective chart review included all patients at a single bloodless institute who refused transfusion between 2003 and 2011. There is no external comparator and the cohort represents a specific subpopulation.	Serious: No control group; associations between care and outcomes were explored using unadjusted logistic regression. Confounding by indication (severity, comorbidities) cannot be accounted for.	Low: Intervention (transfusion-free program) was inherent to the institute and clearly described.	Low: All patients were managed according to the transfusion-free protocol, reflecting a strong patient preference; deviations from intended intervention were unlikely.	Moderate: Outcomes (mortality, hemoglobin levels) were extracted from records; rebleeding and LOS were not comprehensively reported.	Moderate: Retrospective chart review; no information on missing data or loss to follow-up.	Moderate: The study focused on mortality and hemoglobin outcomes, while rebleeding and LOS were incompletely reported; selective reporting cannot be ruled out.	Serious: Lack of comparator and retrospective design mean the study is descriptive. Conclusions about effectiveness are limited; however, the study provides valuable observational data on transfusion-free management.

### 2.6 Data synthesis

Given the diversity of interventions, populations and outcome measures, a quantitative meta-analysis was not appropriate. Instead, findings were summarized narratively and presented in tables (Tables 1, 2). The direction and magnitude of effects were considered alongside methodological quality. Where possible, adjusted estimates from multivariable analyses were prioritized.

**Table 2:** Characteristics of included studies evaluating organizational models for acute gastrointestinal bleeding.

Study (First author, year)	Country/Setting	Study design	Intervention category	Target population	Comparator
Marmo et al., 2021 (24)	Italy; 50 hospitals participating in a regional network; 3,324 patients	Prospective multicenter cohort	Hub-and-spoke network; regionalized triage/transfer system for GI bleeding	Adults with acute upper GI bleeding (variceal and non-variceal)	Hospitals within vs. outside the network
Wang et al., 2021 (25)	China; Third Affiliated Hospital of Nantong University; 206 patients	Retrospective single-center cohort	MDTC; coordinated care by gastroenterologists, nurses and other specialists	Adults with cirrhosis and upper GI bleeding	Traditional care (July 2015–July 2017) vs. MDTC (Aug 2017-Sept 2019)
Sharma et al., 2015 (14)	United States; Englewood Hospital & Medical Center (Bloodless Medicine Institute); 96 patients	Retrospective single-center observational cohort	Transfusion-free management program; structured protocol for patients refusing blood products; multidisciplinary team involvement	Adults with gastrointestinal bleeding (upper or lower) who declined transfusion	None (observational)

### 2.7 Role of the funding source

No external funding was received for this review. The authors conducted the study independently.

### 2.8 Ethical considerations

As this study synthesized data from published research, ethical approval was not required. All included studies had obtained appropriate ethical approval as reported in their original publications.

### 2.9 Reporting guideline adherence

The review was reported in accordance with the PRISMA 2020 statement, which provides updated guidance for transparent reporting of systematic reviews (20). The checklist and flow diagram were completed to ensure all relevant items were addressed.

## 3 Results

### 3.1 Literature search and study selection

A comprehensive search of *PubMed*, *Scopus*, *Embase* and *Web of Science* from 2015 to 2025 identified 978 records. After removing 286 duplicates, 692 unique titles and abstracts were screened. Six hundred eighty-nine records were excluded for reasons such as unrelated topic, narrative or guideline articles, procedural or pharmacologic interventions, case reports or small case series, or insufficient detail. Three full-text articles met the prespecified eligibility criteria and were included in the qualitative synthesis. Figure 1 (PRISMA flow diagram) provides a visual summary of the search process.

#### 3.1.1 Risk-of-bias assessment

**Figure 2:**
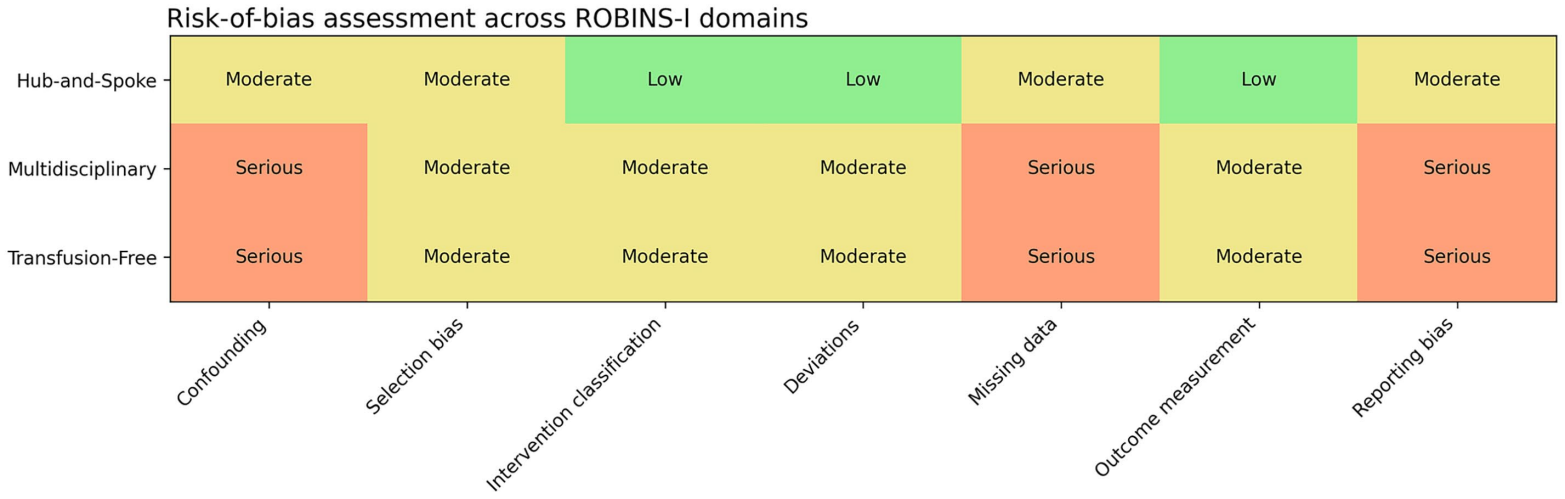
Domain-specific ROBINS-I risk-of-bias plot for included organizational care models in acute gastrointestinal bleeding.

Risk of bias was evaluated using established domains, including selection bias, confounding, classification of intervention, outcome measurement, missing data and overall judgment (Table 1). In addition, a traffic-light plot summarizing domain-specific risk-of-bias judgments is presented in Figure 2. The hub-and-spoke cohort had moderate selection bias due to voluntary hospital participation and moderate confounding despite multivariable adjustment; outcome measurement and classification were low risk, and missing data were incompletely reported. The multidisciplinary team study was vulnerable to selection bias and temporal confounding inherent in before-after designs, with moderate issues in outcome measurement and serious attrition bias owing to differential follow-up. The MDT study’s before-after design is prone to temporal confounding; improvements observed after implementation may stem from contemporaneous advances in cirrhosis management, endoscopic techniques or critical care rather than the MDT itself (22, 23). Although the authors used multivariable regression, residual confounding remains a concern. The transfusion-free cohort lacked a comparator group and relied on retrospective chart review; selection bias was moderate, confounding could not be fully addressed, and missing data were not reported, limiting causal inference. Overall, all studies were judged at moderate or serious risk of bias, underscoring the need for cautious interpretation. For each study, Table 1 summarizes ROBINS-I judgments by domain. As shown in Figure 2, the hub-and-spoke study had moderate risk due to confounding and selection bias but low risk for outcome measurement and classification. The MDT and transfusion-free studies exhibited serious risk in several domains, particularly confounding and missing data, because of their observational designs and lack of concurrent controls.

### 3.2 Characteristics of included studies

Three non-randomized observational studies evaluated organizational or care-delivery models for acute gastrointestinal bleeding. All participants were adults (≥18 years) presenting with upper or lower gastrointestinal hemorrhage in hospital-based settings. The interventions, populations and comparators varied (Table 2).

#### 3.2.1 Hub-and-spoke network (24)

A prospective multicenter cohort study in Italy enrolled 3,324 patients with acute UGIB across 50 hospitals. Of these, 1977 were treated in hospitals participating in a regional hub-and-spoke network that coordinated triage and transfer for digestive emergencies; 1,347 were managed in hospitals outside the network. Patients were managed according to local practice, and outcomes were recorded prospectively. The primary comparison was network versus non-network hospitals (24).

#### 3.2.2 Multidisciplinary team care (25)

This retrospective before-after cohort from China included 206 cirrhotic patients with UGIB managed at a single tertiary hospital between July 2015 and December 2019. Patients admitted before August 2017 received traditional care, whereas those admitted afterwards were managed by a multidisciplinary team composed of gastroenterologists, nurses and other specialists. The study compared one-year mortality, rebleeding and quality of life between the traditional care (*n* = 101) and multidisciplinary team (*n* = 105) cohorts (25). In this study, the authors stated that patients admitted after August 2017 were managed according to a standardized protocol with involvement of gastroenterologists, hepatologists, anesthetists, nurses and dietitians. However, the report did not specify the proportion of patients who received complete MDT evaluation, the timing of consultations or adherence to each component of the pathway. Because implementation fidelity was not reported, the intensity and consistency of MDT care cannot be fully assessed.

#### 3.2.3 Transfusion-free management program (14)

A retrospective chart review from a bloodless medicine institute in the United States examined 96 patients with overt gastrointestinal bleeding who refused blood products between 2003 and 2011. Patients received a structured transfusion-free protocol involving hemostatic optimization, iron supplementation and, when necessary, surgical intervention. There was no external control group (14).

### 3.3 Patient outcomes

#### 3.3.1 Mortality

In the hub-and-spoke study, crude mortality was 5.2% in network hospitals and 6.1% in non-network hospitals; the difference was not statistically significant (Table 3). Multivariable analysis showed that admission to a gastroenterology ward (odds ratio 0.61, *p* = 0.001) or an academic hospital (OR 0.65, *p* = 0.056) were protective factors, whereas network membership itself had no effect on mortality.

**Table 3:** Clinical outcomes of organizational interventions in acute gastrointestinal bleeding.

Study	Mortality outcome	Length-of-stay outcome	Rebleeding outcome	Discharge & resource use	Predictors & risk factors	Effect summary
Marmo et al., 2021 (24)	Mortality 6.1% in non-network hospitals vs. 5.2% in network hospitals; difference not significant.	Mean LOS ≈ 10 days in both groups (10.1 vs. 9.9 days).	Non-variceal rebleeding 4.0% (non-network) vs. 5.6% (network); variceal rebleeding 6.3% vs. 11.0% (higher in network hospitals).	Network hospitals discharged patients directly from the emergency department more often (8.2% vs. 2.6%, *p* < 0.001); surgery and interventional radiology use were similar between groups.	Survival improved when patients were treated in academic hospitals or dedicated gastroenterology wards; network membership itself had no protective effect.	The hub-and-spoke network did not reduce mortality, LOS or surgery rates and had higher rebleeding in both variceal and non-variceal bleeding; improvements were driven by care in academic hospitals/specialized wards.
Wang et al., 2021 (25)	MDTC lowered one-year mortality; multivariable regression identified MDTC as a protective factor against death.	Not reported.	MDTC lowered one-year rebleeding and was protective in multivariable analysis.	Units of packed red blood cells were fewer in the MDTC group; higher transfusion requirement was associated with higher mortality and rebleeding.	Higher MELD and Child-Pugh scores, greater numbers of packed RBC units and lower platelets were risk factors for death or rebleeding; MDTC was protective.	Implementing MDTC for cirrhotic UGIB reduced both mortality and rebleeding and significantly improved quality of life versus traditional care; the benefit persisted after adjusting for disease severity and transfusion needs.
Sharma et al., 2015 (14)	Overall mortality 10.4%; among patients with Hb ≤ 6 g/dL, 79.4% survived; some patients with Hb < 3 g/dL survived.	LOS was treated as a predictor: each additional hospital day increased the odds of death slightly (OR 1.04 per day, *P* = 0.099); mean LOS not reported.	Rebleeding not numerically reported; authors note men had higher rebleeding and surgery rates.	Nearly half the patients (49%) were transfers; 11% presented with haemodynamic instability; surgical interventions were pursued more often because transfusions were not possible.	Logistic regression showed age, ICU admission and anticoagulation use increased mortality; higher initial and nadir hemoglobin were protective; male sex and lower hemoglobin were associated with more rebleeding and need for surgery.	A transfusion-free protocol achieved mortality comparable with conventional care despite refusal of blood products; mortality was influenced more by age, ICU admission and anticoagulation than by the protocol itself.

The multidisciplinary team study reported lower one-year mortality in the team-managed group compared with traditional care. Multivariable logistic regression identified multidisciplinary care as a protective factor, whereas higher the Model for End-Stage Liver Disease (MELD) and Child-Pugh scores and greater transfusion requirements were associated with increased mortality.

In the transfusion-free program, the overall mortality rate was 10.4%. Survival remained high even among patients with very low hemoglobin concentrations; 81% of those with hemoglobin ≤6 g/dL and 57% with hemoglobin <3 g/dL survived. In unadjusted logistic models, older age, intensive care admission and anticoagulation use increased mortality risk, whereas higher initial and nadir hemoglobin were protective.

#### 3.3.2 Rebleeding

Non-variceal rebleeding occurred in 4.0% of patients treated outside the network and 5.6% within the network, while variceal rebleeding was 6.3 and 11.0%, respectively, in Marmo et al.’s study; these differences favored non-network hospitals and were driven by care in academic units rather than network membership (Table 3). In Wang et al., rebleeding within 1 year was significantly reduced in the multidisciplinary care cohort, and multidisciplinary care remained a protective factor after adjusting for disease severity and transfusion requirements. Rebleeding rates were not numerically reported in the transfusion-free study; however, male sex and lower hemoglobin were associated with higher rebleeding and surgical intervention.

#### 3.3.3 Length of stay and resource use

Mean hospital stay was about ten days in both groups of the hub-and-spoke study (10.1 days versus 9.9 days, Table 3). Network hospitals discharged a greater proportion of patients directly from the emergency department (8.2% vs. 2.6%, *p* < 0.001), but surgical and interventional radiology use were similar. In the transfusion-free cohort, length of stay was treated as a predictor rather than an outcome; each additional hospital day modestly increased mortality risk (OR 1.04 per day, *p* = 0.099). Length of stay was not reported in the multidisciplinary team study, although the intervention was associated with fewer units of packed red blood cells and improvements in quality-of-life scores.

#### 3.3.4 Summary and synthesis

The available evidence suggests that organizational models can influence outcomes in acute gastrointestinal bleeding (Table 3). The Italian hub-and-spoke network did not reduce mortality or rebleeding relative to usual care, and improvements were largely attributable to treatment in academic centers or dedicated gastroenterology wards. In contrast, multidisciplinary team care for cirrhotic UGIB reduced both mortality and rebleeding at 1 year and improved patient-reported quality of life. Wang et al. reported that baseline characteristics, including age, sex, etiology of cirrhosis, Child-Pugh class, MELD score, presence of active bleeding and need for rescue therapy, were similar between the traditional care and MDT groups. Their multivariable regression model adjusted for MELD, Child-Pugh score and transfusion requirements, and MDT care remained a protective factor for mortality. Nevertheless, residual confounding cannot be excluded. Outcomes after variceal bleeding are strongly influenced by severity scores (26), active bleeding status and the use of rescue treatments; any imbalance in unmeasured variables could have biased the results. A transfusion-free management program achieved mortality comparable with conventional care despite refusal of blood products, with survival influenced more by age, critical illness and anticoagulation use than by the protocol itself. Because of heterogeneity in interventions and outcomes, meta-analysis was not performed.

## 4 Discussion

### 4.1 Principal findings: comparison with existing evidence

This review synthesized evidence from three observational studies evaluating organizational strategies for acute gastrointestinal bleeding. We found that a regional hub-and-spoke network for UGIB did not significantly reduce mortality or rebleeding compared with care provided outside the network. In contrast, MDT care for cirrhotic UGIB was associated with a reduction in one-year mortality and rebleeding and improvements in quality of life. A structured transfusion-free protocol for patients who refused blood products achieved survival rates comparable to conventional care, although outcomes were heavily influenced by patient age, severity of illness and need for intensive care. Overall, the evidence suggests that organizational models can affect patient outcomes, but the direction and magnitude of effect depend on the specific intervention and context.

The hub-and-spoke network study enrolled more than 3,000 patients across 50 hospitals and represents the largest cohort of organizational care for UGIB. It’s finding that network membership itself did not improve survival contrasts with the theoretical benefits of regionalization. In trauma, stroke and myocardial infarction, hub-and-spoke systems have reduced mortality through centralization of expertise and resources. However, acute GIB requires timely endoscopy, which can often be delivered locally; delays in transfer may negate the benefits of specialized centers. The study observed that admission to academic hospitals or dedicated gastroenterology wards, rather than network affiliation, was protective. This aligns with research showing that variations in hospital performance are more closely related to institutional capability and staffing than network structure (12, 27). In the absence of evidence that centralization improves outcomes, policymakers should focus on ensuring adequate specialist staffing and training at all hospitals.

The MDT care study reported reductions in one-year mortality and rebleeding for cirrhotic patients, supporting the premise that complex GIB benefits from coordinated multidisciplinary management. Cirrhotic bleeding is complicated by portal hypertension, coagulopathy and multiorgan dysfunction; an MDT can simultaneously address haemodynamic resuscitation, pharmacologic therapy, endoscopic variceal control and post-procedure monitoring. These findings mirror those from a trauma center where a multidisciplinary communication protocol shortened time to endoscopy, reduced transfusion requirements and decreased hospital length of stay (3). Similarly, the BSG care bundle emphasizes early risk stratification and call-for-help protocols to ensure timely specialist input (8, 9). Collectively, these data suggest that structured communication and role delineation, rather than simply the presence of multiple disciplines, underpin the success of MDT interventions. Because the MDT study compared patients from different time periods, secular improvements in the management of portal hypertension, endoscopic variceal therapy and intensive care support may partly explain the observed reduction in mortality and rebleeding. Randomized or stepped-wedge trials are needed to determine causality (22, 23).

The transfusion-free management program demonstrated that mortality remained around 10% despite patients refusing blood products. Survival was high even at hemoglobin concentrations <6 g/dL, supporting restrictive transfusion strategies endorsed by guidelines (28). By employing intravenous iron, erythropoietin and meticulous haemostatic control, the program avoided transfusion without increasing mortality, echoing evidence from prospective trials showing that transfusion thresholds of 7 g/dL are safe and may reduce rebleeding. However, the absence of a comparator group and small sample size limit generalisability. Notably, older age, intensive care admission and anticoagulation use predicted mortality, emphasizing that patient factors remain the primary determinants of outcome even in the context of innovative service models (28, 29).

### 4.2 Interpretation of organizational models

The lack of benefit from the hub-and-spoke model could reflect several factors (24). First, timely endoscopy is critical; guidelines recommend performing endoscopy within 24 h of presentation for most patients (30, 31). Transfer to a hub may delay intervention, whereas network hospitals with on-call endoscopists can deliver care promptly (31). Second, the network did not standardize treatment protocols or risk stratification; variation in practice across sites may have masked any potential advantage (31, 32). Third, the network lacked dedicated funding to enhance staffing or training, so network membership alone may not overcome baseline differences in resources (33). Finally, confounding by patient severity is possible; more severe cases may have been preferentially transferred to the hub, biasing results toward the null (31, 34).

For MDT care, benefits likely stem from comprehensive assessment and coordination (35, 36). Early involvement of hepatologists ensures optimization of portal hypertension management with vasoactive medications, while anesthetists can guide haemodynamic resuscitation (37). Nurses trained in liver disease provide targeted education and ensure adherence to secondary prophylaxis (36). The observed reductions in mortality and rebleeding in the MDT study are consistent with reports from other chronic diseases, where interdisciplinary team care improves outcomes (35, 36). However, the before-after design introduces temporal confounding; improvements could also reflect general advances in endoscopy or pharmacotherapy (38). Furthermore, the study included only cirrhotic patients, limiting applicability to the broader GIB population. Nevertheless, the magnitude of benefit supports investment in MDT programs, particularly in tertiary centers managing complex cases (35, 36).

Transfusion-free protocols showcase the feasibility of managing GIB without blood products (39). By adopting a restrictive transfusion threshold (hemoglobin <7 g/dL) and focusing on haemostatic optimization, such programs align with international guidelines and may reduce transfusion-related complications (28, 29, 40). Evidence from large randomized trials suggests that a restrictive strategy decreases mortality and rebleeding compared with liberal transfusion in variceal bleeding (40). Nevertheless, the bloodless institute study lacks a control group and may overestimate survival due to selection bias; patients willing to undergo transfusion-free care may differ systematically from general GIB populations (34, 39). The program also involved a multidisciplinary team and dedicated resources, raising questions about cost-effectiveness and scalability (39). Future studies should compare transfusion-free protocols with standard care in prospective designs, assessing patient-centered outcomes and resource utilization (40).

### 4.3 Organizational factors beyond the included studies

While our review focused on three interventions, other organizational strategies merit discussion. Care bundles, such as those recommended by the BSG, combine evidence-based steps including early resuscitation, risk stratification, proton pump inhibitor therapy and timely endoscopy (8). A multicenter British audit found that implementation of a care bundle improved adherence to guideline components and was associated with lower mortality compared with historical cohorts (8). Similarly, care bundles in sepsis and acute kidney injury have improved outcomes, suggesting that structured checklists can standardize care and reduce variability. Another organizational factor is risk stratification. Tools such as the GBS, AIMS65 and Rockall scores assist in triaging patients to outpatient management or intensive care (6). Accurate risk assessment may allow safe discharge of low-risk patients, reducing hospital admissions and freeing resources for high-risk cases. Emerging risk scores incorporating lactate, base deficit and machine-learning approaches show promise (6, 41). Finally, hospital performance metrics highlight variation in outcomes; only a minority of hospitals improve mortality and length of stay simultaneously (12, 42). Benchmarking and feedback may encourage adoption of best practices across institutions.

### 4.4 Implications for practice and policy

Several implications arise from our findings. First, policymakers should be cautious in assuming that regional hub-and-spoke networks will automatically improve GIB outcomes; without investment in specialist staffing, endoscopy availability and protocols, network membership alone may not confer benefit. Instead, efforts should focus on ensuring that all hospitals can provide early resuscitation and endoscopy, with clear pathways for referral of complex cases. Second, hospitals managing high volumes of cirrhotic or complex GIB should consider establishing MDT programs. Evidence suggests that coordinated care reduces mortality and rebleeding (3, 43), and such programs align with guidelines calling for early specialist involvement (8). Third, the success of transfusion-free protocols highlights the importance of restrictive transfusion thresholds; even outside bloodless medicine units, clinicians should adhere to guideline-recommended hemoglobin triggers and optimize hemostasis (28, 29). Finally, implementation of care bundles and risk stratification tools may standardize practice, improve documentation and facilitate auditing. Institutions should train staff on scoring systems and embed checklists into electronic health records.

### 4.5 Strengths and limitations of this review

This review offers a comprehensive synthesis of the evidence on organizational interventions for acute gastrointestinal bleeding, an area that has received relatively little attention compared with pharmacologic or endoscopic strategies. We implemented a structured search across multiple databases with guidance from an information specialist, applied clear eligibility criteria, and conducted independent dual screening, data extraction, and risk-of-bias assessment using the ROBINS-I tool.

Nevertheless, several limitations warrant consideration. Only three studies met inclusion criteria, reflecting the scarcity of research in this domain. All included studies were observational, non-randomized, and at moderate to serious risk of bias, with potential confounding, selection bias, and missing data affecting the reliability of findings. The heterogeneity of interventions and outcomes prevented meta-analysis, and the small sample of studies limited opportunities for subgroup or sensitivity analyses. Additionally, publication bias may be present, as studies demonstrating null or negative effects of organizational models may remain unpublished. Despite these constraints, the review provides valuable insights into different approaches regional networks, multidisciplinary teams, and transfusion-free programs and offers a conceptual framework to guide future research and policy decisions. The identification of only three eligible studies underscores how under-researched organizational care models are; our conclusions should therefore be viewed as hypothesis-generating rather than definitive. This paucity of evidence highlights an urgent need for well-designed prospective studies to determine whether structural interventions improve outcomes. One more thing that must be noticed, although the MDT study attempted to adjust for disease severity and transfusion requirements, small sample size and lack of detailed reporting on active bleeding or need for salvage therapies limit confidence in the findings. Since Child-Pugh and MELD scores are strong predictors of mortality (26), even modest imbalances can affect outcomes. Moreover, the MDT study did not describe how many patients actually received all elements of the protocol or how adherence was monitored, limiting our ability to gauge implementation fidelity. Without such data, it is unclear whether the observed benefits were due to full MDT involvement or to other contemporaneous improvements. Finally, our search was restricted to English-language articles and may have missed relevant studies in other languages. In addition, the protocol was not registered; although the methods were specified in advance, the lack of registry entry may reduce transparency.

### 4.6 Future research directions

Future studies should priorities rigorous evaluation of organizational interventions (44, 45). Randomized controlled trials or well-designed quasi-experimental studies (e.g., stepped-wedge cluster trials) could test hub-and-spoke networks, care bundles, MDT programs and transfusion-free protocols (46). Trials should incorporate process measures (time to endoscopy, adherence to bundle elements) and patient-centered outcomes (mortality, rebleeding, quality of life) (44). Risk stratification tools should be validated prospectively in diverse populations and integrated into decision-support systems (47, 48). Economic evaluations are needed to assess cost-effectiveness of organizational models, including resource utilization, staff training and infrastructure costs (49–51). Qualitative research exploring barriers and facilitators to implementation could inform adaptation across settings (19). Finally, future systematic reviews should update the evidence as more studies emerge and explore the interplay between organizational factors and clinical innovations (52, 53).

## 5 Conclusion

Careful synthesis of the three available studies suggests that the way hospitals organize services for acute gastrointestinal bleeding might influence patient outcomes, yet the current evidence base is too limited to draw firm conclusions. A regional hub-and-spoke network did not demonstrate any reduction in mortality compared with non-network hospitals, implying that simply affiliating with a network without investing in standardized protocols or additional resources is unlikely to improve outcomes. In contrast, a multidisciplinary team program for cirrhotic variceal bleeding and a transfusion-free pathway within a bloodless medicine center were associated with favorable mortality and utilization outcomes, but both studies were single-center, observational and at moderate-to-serious risk of bias. These preliminary findings should therefore be regarded as hypothesis-generating rather than definitive evidence of effectiveness. Overall, the review underscores the need for robust multicentre trials, economic evaluations and implementation studies to determine whether multidisciplinary care, patient-blood-management programs or regional networks can reliably improve survival, reduce rebleeding and optimize resource use for patients with acute gastrointestinal bleeding.

## Data Availability

The datasets presented in this study can be found in online repositories. The names of the repository/repositories and accession number(s) can be found at: 10.5281/zenodo.20139704.
