# Supplementary material for: Organizational models for acute gastrointestinal bleeding: a systematic review of hospital networks, multidisciplinary care and bloodless programs (2015–2025)
Source: Front Med (Lausanne). 2026 Jul 2;13:1881585. doi: 10.3389/fmed.2026.1881585 (PMC13372989; doi:10.3389/fmed.2026.1881585)
Supplement: Supplementary file 1 [file Table_1.DOCX]

Supplementary Material

# Table S1. PubMed Search Strategy for Studies on Organizational Models and Care Delivery in Acute Gastrointestinal Bleeding

| **No** | **Query (PubMed syntax)** |
| --- | --- |
| 1 | "Gastrointestinal Hemorrhage"[MeSH] OR ("gastrointestinal hemorrhage"[tiab] OR "gastrointestinal bleeding"[tiab] OR "GI bleeding"[tiab] OR "upper GI bleed"[tiab] OR "lower GI bleed"[tiab] OR hematemesis[tiab] OR melena[tiab]) |
| 2 | "Delivery of Health Care"[MeSH] OR ("delivery of health care"[tiab] OR "healthcare delivery"[tiab] OR "service delivery"[tiab] OR "organization of care"[tiab] OR organiz* of health care[tiab]) |
| 3 | "Patient Care Team"[MeSH] OR "Critical Pathways"[MeSH] OR ("care pathway"[tiab] OR "clinical pathway"[tiab] OR "critical pathway"[tiab] OR "care coordination"[tiab] OR "multidisciplinary team"[tiab] OR "care model"[tiab]) |
| 4 | #2 OR #3 |
| 5 | #1 AND #4 |
| 6 | Limits: Humans; Publication date 2015/01/01:2025/12/31; English language; Age≥19; Free full text |

# Scopus Search Strategy for Identification of Organizational and Multidisciplinary Care Studies in Gastrointestinal Hemorrhage

| **No** | **Query (Scopus syntax)** |
| --- | --- |
| 1 | "gastrointestinal hemorrhage" OR "gastrointestinal bleeding" OR "GI bleeding" OR hematemesis OR melena OR "upper gastrointestinal bleed" OR "lower gastrointestinal bleed" |
| 2 | "delivery of health care" OR "healthcare delivery" OR "service delivery" OR organization* OR "care pathway" OR "clinical pathway" OR "critical pathway" OR "care coordination" OR "multidisciplinary team" OR "care model" |
| 3 | 1 AND 2 |
| 4 | Limit: PUBYEAR > 2015 AND PUBYEAR < 2025; Human subject area; English language; All open access |

# Web of Science Search Strategy for Literature on Health Care Delivery Models in Acute Gastrointestinal Bleeding

| **No** | **Query (Web of Science syntax)** |
| --- | --- |
| 1 | TS=( "gastrointestinal hemorrhage" OR "gastrointestinal bleeding" OR "GI bleeding" OR hematemesis OR melena OR "upper GI bleed" OR "lower GI bleed" ) |
| 2 | TS=( "delivery of health care" OR "healthcare delivery" OR "service delivery" OR organization* OR "care pathway" OR "clinical pathway" OR "critical pathway" OR "care coordination" OR "multidisciplinary team" OR "care model" ) |
| 3 | TS=( "mouse" OR "mice" OR "rat" OR "rats" OR "rabbit*" OR "dog" OR "dogs" OR "cat" OR "cats" OR "pig" OR "pigs" OR "swine" OR "murine" OR "rodent*" OR "animal*") |
| 3 | #1 AND #2 NOT #3 |
| 4 | Refine: Years 2015–2025; Document types (Article, Review, Guideline, Conference Paper if relevant); Languages as required |

# Embase (Emtree and Keyword) Search Strategy for Organizational Interventions in Gastrointestinal Bleeding Management

| **No** | **Query (Emtree + Keywords)** |
| --- | --- |
| **1** | exp gastrointestinal hemorrhage |
| **2** | ("gastrointestinal hemorrhage" OR "gastrointestinal bleeding" OR "GI bleed*" OR "GI hemorrhage" OR hematemesis OR melena OR "upper GI bleed" OR "lower GI bleed").ti,ab,kw. |
| **3** | 1 OR 2 |
| **4** | exp health care delivery |
| **5** | (healthcare delivery OR "delivery of health care" OR "service delivery" OR "organization of care" OR "organizational model*" OR reorganize* OR redesign*).ti,ab,kw. |
| **6** | exp clinical pathway OR exp critical pathway |
| **7** | ("clinical pathway" OR "critical pathway" OR "care pathway" OR "care protocol*" OR "care coordination" OR "multidisciplinary team*" OR "team-based care" OR "care model*" OR "bleeding pathway").ti,ab,kw. |
| **8** | 4 OR 5 OR 6 OR 7 |
| **9** | 3 AND 8 |
| **10** | Limit 9 to yr="2015 - 2025" |
| **11** | Limit 10 to humans |
| **12** | Limit 11 to 18 Y/O above |
| **13** | Limit 12 to English |
